# The diagnostic value of serum inflammation, coagulation and tumor indexes in differentiating stage III and IV ovarian endometriotic cysts

**DOI:** 10.3389/fmed.2025.1733063

**Published:** 2026-01-12

**Authors:** Li-Juan Lu, Xue-Fei Liu, Yan-Yan Xing

**Affiliations:** 1 Department of Gynecology and Obstetrics, Jinshan Hospital of Fudan University, Shanghai, China; 2 Department of Radiology, Jinshan Hospital of Fudan University, Shanghai, China

**Keywords:** endometriosis staging, integration of PLR, FIB, and CA125, nomogram, ovarian endometriotic cysts, stage

## Abstract

**Background:**

Ovarian endometriotic cysts (OEC) are classified into early (I-II) and advanced (III-IV) stages. Stage IV OEC is characterized by severe adhesions and anatomical distortion that complicate surgical management. Accurate preoperative differentiation between stage III and IV OEC is crucial for optimizing surgical planning and patient outcomes.

**Objective:**

This study aimed to investigate the diagnostic value of serum inflammatory, coagulation, and tumor markers in differentiating stage III from stage IV OEC.

**Methods:**

This retrospective study analyzed 205 patients with histopathologically confirmed stage III (*n* = 132) and stage IV (*n* = 73) OEC who underwent surgical treatment between January 2013 and December 2019. Patients were divided into training (70%) and test (30%) cohorts. Inflammatory markers, coagulation parameters, and tumor markers were evaluated. Multivariate logistic regression identified independent predictors, and a nomogram was constructed to predict stage IV disease.

**Results:**

Stage IV patients demonstrated significantly elevated platelet-to-lymphocyte ratio (PLR), thrombin time, fibrinogen (FIB) levels, and carbohydrate antigen 125 (CA125) concentrations compared to stage III patients. Multivariate analysis identified PLR, FIB, and CA125 as independent predictors of stage IV OEC. The combined nomogram (PLR, FIB, and CA125) achieved superior diagnostic performance with an AUC of 0.81 (95% CI: 0.75–0.88) in the training cohort and 0.78 (95% CI: 0.65–0.91) in the test cohort, outperforming individual biomarkers.

**Conclusion:**

The integration of PLR, FIB, and CA125 provides a practical, non-invasive, and cost-effective approach for preoperative differentiation of stage III and IV OEC, which potentially facilitates improved surgical planning and personalized patient management.

## Introduction

Endometriosis is a chronic and estrogen-dependent gynecological disorder, which is characterized by the presence of functional endometrial glands and stroma outside the uterine cavity (1). Endometriosis predominantly affects women of reproductive age and is associated with pelvic pain, dysmenorrhea, dyspareunia, and infertility. Ovarian endometriotic cyst (OEC), also referred to as “chocolate cyst,” represents the most prevalent form of endometriosis (2). OEC leads to the accumulation of hemolyzed blood and hemosiderin. Over time, this process promotes inflammation, fibrosis, and adhesion formation, which results in distortion of pelvic anatomy and impaired fertility.

OEC is typically identified in early stages (I-II) and advanced stages (III-IV) (3). The advanced stages are characterized by the presence of large cysts, dense pelvic adhesions, and extensive anatomical involvement (4). While both stage III and IV OEC are advanced disease, they differ in the degree of fibrosis, pelvic adhesion severity, and ovarian involvement. Stage IV disease is often associated with more severe adhesions, which can complicate surgical excision and increase the risk of intraoperative bleeding (5). Early identification of patients with potential stage IV OEC can therefore assist in optimizing surgical strategy selection and facilitate multidisciplinary management for fertility preservation (6).

Recent research has highlighted the complex interplay among inflammation, coagulation, and tumor marker expression in the pathophysiology of OEC. Chronic inflammation is known to activate the coagulation cascade and platelet aggregation (7). Systemic inflammatory markers such as the neutrophil-to-lymphocyte ratio (NLR) and platelet-to-lymphocyte ratio (PLR) have emerged as potential indicators of endometriosis activity and severity (8). Similarly, alterations in coagulation parameters, including fibrinogen (FIB), thrombin time (TT), and activated partial thromboplastin time (APTT), have been reported in OEC patients (9). Furthermore, tumor markers such as carbohydrate antigen 125 (CA125) and carbohydrate antigen 199 (CA199) are routinely used in the evaluation of ovarian lesions, and their levels have been shown to correlate with OEC stages (10).

Despite these associations, few studies have systematically explored the diagnostic value of integrating serum inflammatory, coagulation, and tumor indexes for distinguishing between stage III and IV OEC. Identifying specific biomarker patterns that correspond to disease severity could enhance preoperative staging accuracy, provide insights into disease progression, and support individualized management strategies. Therefore, the present study aimed to investigate the diagnostic significance of serum inflammatory, coagulation, and tumor markers in differentiating stage III from stage IV OEC.

## Materials and methods

### Study design and ethics considerations

This retrospective study analyzed the clinical and laboratory data of patients who underwent surgical treatment for OEC at the Department of Gynecology and Obstetrics, Jinshan Hospital of Fudan University, between January 2013 and December 2019. The study was approved by the Ethics Committee of Jinshan Hospital of Fudan University (2025–S92), and written informed consent was waived.

### Participants

All patients with histopathologically confirmed OEC (stage III and stage IV) were included in the study. The exclusion criteria were as follows: Coexisting adenomyosis, pelvic inflammatory disease, or pregnancy; Presence of ovarian or other malignancies; Known autoimmune, endocrine, or systemic inflammatory diseases; History of hormonal therapy within 3 months prior to surgery or previous ovarian surgery; Incomplete clinical or laboratory data.

The patients were categorized into two groups: stage III group and stage IV group. Subsequently, the data was randomly split into a training cohort and a test cohort, following a 7:3 ratio.

### Clinical and surgical evaluation

The histopathological diagnostic criteria were based on standard histopathological guidelines from the World Health Organization classification of female reproductive tract tumors. All cases were reviewed independently by two experienced pathologists blinded to clinical and imaging data. Any discrepancies were resolved by consensus discussion. Electronic medical records of all the patients were reached. OEC were classified according to the revised American Society for Reproductive Medicine (rASRM) classification system (1997) into stages III or IV based on operative records. Staging was independently determined by experienced gynecologist and radiologist, and in cases of discrepancy, a third senior gynecologist reviewed the operative record and images to reach consensus. Clinical symptoms including dysmenorrhea, cyst rupture, recurrence and infertility were also documented. The cyst size was determined through histopathological records using the formula of length × width × height × 0.523.

### Laboratory tests

Routine hematological and biochemical analyses were investigated. All blood samples were collected within 1 week before surgery during the early follicular phase. Patients were fasting for at least 8 h prior to blood collection, and samples were drawn in the morning. Inflammatory markers including white blood cell count (WBC), neutrophil count (NE, × 10^9^/L), lymphocyte count (LY, × 10^9^/L), platelet count (PLT, × 10^9^/L), neutrophil-to-lymphocyte ratio (NLR), and platelet-to-lymphocyte ratio (PLR); coagulation parameters including prothrombin time (PT, s), activated partial thromboplastin time (APTT, s), thrombin time (TT, s), fibrinogen (FIB, g/L), international normalized ratio (INR), and plasma D-dimer (mg/L); tumor markers including carbohydrate antigen 125 (CA125, U/mL), carbohydrate antigen 199 (CA199, U/mL), carcinoembryonic antigen (CEA, ng/mL), alpha-fetoprotein (AFP, ng/mL), cancer antigen 153 (CA153, U/mL) and human epididymis protein 4 (HE4, pmol/L) were investigated.

### Predictors selection and nomogram building

Using data from the training cohorts, a univariate binary logistic regression was initially conducted to identify differences between stage III and stage IV OEC. Subsequently, a multivariate binary logistic regression analysis was employed to select the laboratory tests that could predict stage IV OEC. Variables with *P* < 0.05 in univariate binary logistic regression were used as the potential candidates for multivariate modeling. To avoid multicollinearity and overfitting in the predictive model, a systematic variable selection process was performed. Collinearity among inflammatory markers was assessed using Spearman correlation coefficients. Variables with strong correlations (*r* > 0.70) were evaluated. Finally, a nomogram was constructed for predicting stage IV OEC by integrating laboratory predictors through multivariate binary logistic regression analysis with the lowest Akaike information criterion score.

### Nomogram discrimination and calibration

The effectiveness of the nomogram in predicting stage IV OEC was evaluated by calculating the area under the receiver operating characteristic (ROC) curve (AUC) for both the training and test cohorts. Furthermore, the goodness of fit of the nomogram for these datasets was assessed using calibration curves for both the training and test cohorts.

### Clinical usefulness

A clinical decision curve (CDC) analysis was conducted to assess the clinical usefulness of the nomogram, measuring the net benefits it provided at various threshold probabilities. This assessment was applied to both the training and test cohorts.

### Statistical analysis

All statistical analyses were performed using R (version 4.5.1). Continuous variables were tested for normality using the Kolmogorov-Smirnov test. Normally distributed data were compared using the independent-samples *t*-test. Non-normally distributed data were compared using the Mann-Whitney U test for two groups. Categorical variables were analyzed using the chi-square test. Correlations between clinical and laboratory parameters were assessed using Spearman’s correlation coefficient. The train/test split was performed using the “createDataPartition ()” function in the “caret” package of R, with stratified randomization by disease stage (stage III and stage IV) applied. A two-tailed *P* < 0.05 was considered statistically significant.

## Results

### Baseline characteristics of study participants

**FIGURE 1:**
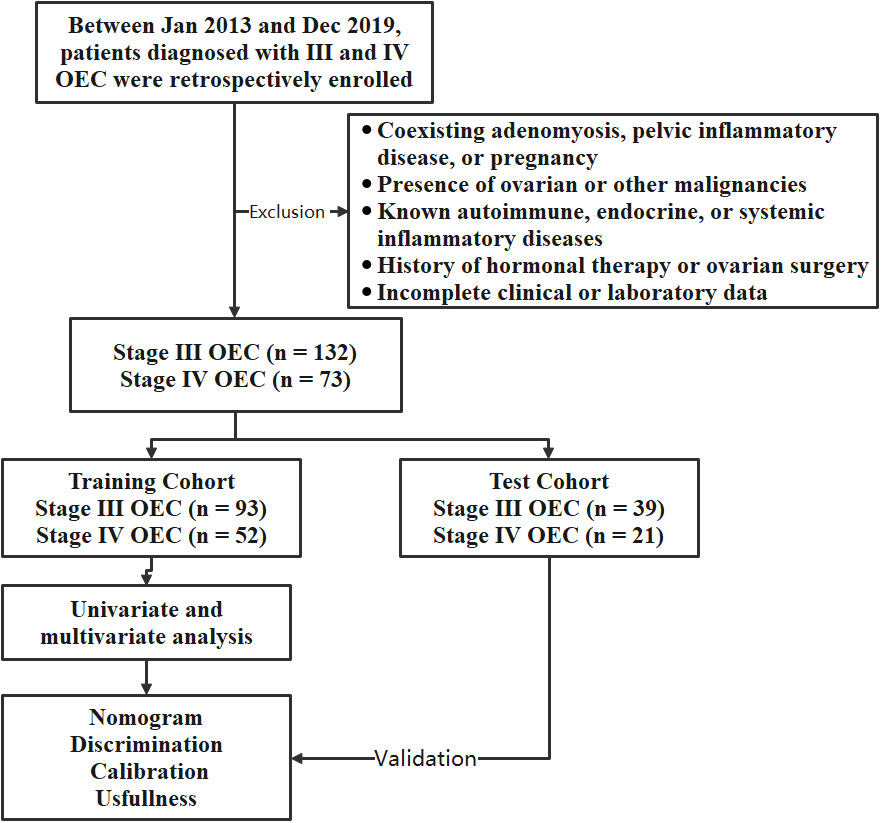
Study workflow and patient selection.

The work flow of this study is shown in Figure 1. Thirteen patients were excluded due to missing laboratory parameters. A total of 205 patients with OEC were included in this study. In the training cohort, 93 patients were stage III and 52 were stage IV. In the test cohort, 39 were stage III and 21 were stage IV. There were no significant differences in age between the stage III and stage IV groups in either the training or test cohorts (*P* = 0.342 and *P* = 0.658, respectively).

Clinical symptoms, including dysmenorrhea, cyst rupture, and recurrence, showed no statistically significant differences between the two stages (all *P* > 0.05). The frequency of pregnancies, deliveries, cesarean sections, and abortions were also similar across groups (*P* > 0.05). The mean cyst volumes were comparable between stage III and stage IV patients in both cohorts (*P* = 0.392 and *P* = 0.614) (Table 1).

**TABLE 1:** Baseline clinical characteristics and laboratory parameters of patients with stage iii and stage IV ovarian endometriotic cysts in training and test cohorts.

Parameters	Training cohort			Test cohort		
	Stage III (*N* = 93)	Stage IV (*N* = 52)	*P*-value	Stage III (*N* = 39)	Stage IV (*N* = 21)	*P*-value
Age (y)	37.6 ± 7.32	38.9 ± 8.40	0.342	37.0 ± 8.11	37.9 ± 7.15	0.658
**Dysmenorrhea**						
Negative	48 (51.6%)	21 (40.4%)	0.261	21 (53.8%)	7 (33.3%)	0.212
Positive	45 (48.4%)	31 (59.6%)		18 (46.2%)	14 (66.7%)	
**Dysmenorrhea degree**						
Mid	13 (14.0%)	10 (19.2%)	0.268	4 (10.3%)	3 (14.3%)	0.315
Mild	30 (32.3%)	17 (32.7%)		14 (35.9%)	11 (52.4%)	
Negative	48 (51.6%)	21 (40.4%)		21 (53.8%)	7 (33.3%)	
Severe	2 (2.2%)	4 (7.7%)		0 (0%)	0 (0%)	
**Rupture**						
Negative	89 (95.7%)	50 (96.2%)	1.000	35 (89.7%)	18 (85.7%)	0.966
Positive	4 (4.3%)	2 (3.8%)		4 (10.3%)	3 (14.3%)	
**Recurrence**						
Negative	86 (92.5%)	50 (96.2%)	0.602	37 (94.9%)	20 (95.2%)	1.000
Positive	7 (7.5%)	2 (3.8%)		2 (5.1%)	1 (4.8%)	
Number of pregnancies (times)	0.2 ± 0.8	0.2 ± 0.8	0.555	0.2 ± 0.7	0.3 ± 0.9	0.609
Number of deliveries (times)	0.1 ± 0.2	0.2 ± 0.6	0.204	0.1 ± 0.4	0.1 ± 0.5	0.782
Cesarean section (times)	0.05 ± 0.22	0.01 ± 0.13	0.258	0.05 ± 0.22	0.04 ± 0.21	0.951
Abortion (times)	0.1 ± 0.6	0.1 ± 0.2	0.680	0.1 ± 0.3	0.1 ± 0.5	0.477
Volume (cm^3^)	177 ± 208	143 ± 242	0.392	174 ± 248	212 ± 288	0.614
WBC (10^9^/L)	6.2 ± 2.07	6.1 ± 1.72	0.789	6.7 ± 1.88	7.1 ± 3.26	0.671
Neutrophils (10^9^/L)	4.2 ± 2.04	4.5 ± 1.55	0.367	4.7 ± 1.66	5.2 ± 3.01	0.491
LY (10^9^/L)	1.6 ± 0.49	1.3 ± 0.45	0.001	1.5 ± 0.50	1.4 ± 0.52	0.342
PLT (10^9^/L)	223 ± 51.2	254 ± 53.5	0.001	207 ± 54.8	252 ± 51.0	0.003
NLR	3.1 ± 2.7	3.8 ± 1.7	0.064	3.4 ± 1.78	4.0 ± 2.54	0.325
PLR	151 ± 52.1	214 ± 72.3	< 0.001	145 ± 47.3	195 ± 58.8	0.002
PNR	63 ± 35.9	61 ± 21.7	0.649	49 ± 22.5	57 ± 21.8	0.191
PT (s)	11.3 ± 0.94	11.6 ± 1.10	0.155	11.4 ± 1.09	11.2 ± 1.45	0.637
INR	1.01 ± 0.07	1.0 ± 0.09	0.308	1.0 ± 0.08	1.0 ± 0.11	0.740
APTT (s)	32.7 ± 4.08	32.0 ± 3.77	0.296	31.9 ± 2.86	31.7 ± 3.93	0.878
FIB (g/L)	3.0 ± 0.67	3.5 ± 0.75	< 0.001	3.1 ± 0.54	3.5 ± 0.82	0.051
Thrombin time (s)	14.4 ± 1.42	15.1 ± 1.58	0.017	14.7 ± 1.34	14.2 ± 1.67	0.228
D-dimer (mg/L)	0.2 ± 0.17	0.2 ± 0.23	0.038	0.2 ± 0.20	0.3 ± 0.23	0.288
Antithrombin activity (%)	102 ± 11.7	103 ± 12.5	0.867	98.1 ± 14.3	106 ± 15.1	0.072
CA125 (U/mL)	45.9 ± 33.7	89.1 ± 81.6	0.001	50.0 ± 43.3	78.7 ± 65.4	0.081
CA153 (U/mL)	6.7 ± 3.3	7.6 ± 3.8	0.172	8.7 ± 4.8	8.8 ± 3.4	0.992
CA199 (U/mL)	21.2 ± 30.3	28.6 ± 35.3	0.203	31.6 ± 54.6	44.3 ± 71.8	0.486
CEA (ng/mL)	1.0 ± 0.5	0.9 ± 0.5	0.204	1.1 ± 0.54	0.9 ± 0.4	0.139
AFP (ng/mL)	2.2 ± 1.0	2.2 ± 1.0	0.994	2.3 ± 0.8	2.3 ± 1.1	0.990
HE4 (pmol/L)	44.6 ± 8.2	42.4 ± 8.8	0.153	43.7 ± 7.3	44.3 ± 8.0	0.805

AFP, alpha-fetoprotein; APTT, activated partial thromboplastin time; CA125, carbohydrate antigen 125; CA153, carbohydrate antigen 153; CA199, carbohydrate antigen 199; CEA, carcinoembryonic antigen; FIB, fibrinogen; HE4, human epididymis protein 4; INR, international normalized ratio; LY, lymphocytes; NLR, neutrophil-to-lymphocyte ratio; PLR, platelet-to-lymphocyte ratio; PLT, platelet count; PNR, platelet-to-neutrophil ratio; PT, prothrombin time; TT, thrombin time; WBC, white blood cell count. Data are presented as mean ± standard deviation or n (%).

### Comparison of inflammatory markers

Among inflammatory parameters, lymphocyte counts were significantly lower in stage IV compared with stage III patients in the training cohort (*P* = 0.001), while platelet counts were markedly higher in stage IV patients (*P* = 0.001). In the test cohort, the platelet levels remained significantly elevated in stage IV patients (*P* = 0.003).

The PLR was significantly higher in stage IV patients in both the training (*P* < 0.001) and test cohorts (*P* = 0.002). NLR was higher in stage IV disease but only reached marginal statistical significance in the training cohort (*P* = 0.064). WBC and PNR also showed no significant differences between groups (*P* > 0.05) PLR were prioritized over PLT and LY as it represents integrated immune responses (Table 1).

### Comparison of coagulation parameters

Regarding coagulation markers, FIB levels were significantly elevated in stage IV compared with stage III patients in the training cohort (*P* < 0.001), with a similar trend in the test cohort (*P* = 0.051). TT was longer in stage IV patients in the training cohort (*P* = 0.017), but no significant difference was observed in the test cohort (*P* = 0.228). D-dimer levels were higher in stage IV patients in the training cohort (*P* = 0.038), while PT, INR, APTT, and antithrombin activity did not differ significantly between groups (all *P* > 0.05) (Table 1).

### Comparison of tumor markers

CA125 levels were significantly higher in stage IV compared with stage III patients in the training cohort (*P* = 0.001). In the test cohort, CA125 showed an increasing trend but only reached a marginal statistical significance (*P* = 0.081). Other tumor markers, including CA153, CA199, CEA, AFP, and HE4 showed no significant differences between stages in either cohort (*P* > 0.05) (Table 1).

### Multivariate analysis and correlation analysis

To enhance clinical interpretability, PLR and CA125 were scaled per 10-unit increase, and FIB per 1 g/L increase, when calculating odds ratios in the multivariable logistic regression model. Multivariate logistic regression analysis identified PLR, FIB, and CA125 as independent predictors of stage IV OEC in the training cohort (Table 2). Specifically, PLR (range: 100–200, OR = 1.05, 95% CI: 1.01–1.10, *P* = 0.023), FIB (range: 2.0–4.0 g/L, OR = 1.11, 95% CI: 1.02–1.21, *P* = 0.019), and CA125 (range: 0–35 U/mL, OR = 1.03, 95% CI: 1.00–1.06, *P* = 0.036) were significantly associated with an increased likelihood of stage IV disease. Age, lymphocyte count, platelet count, NLR, and D-dimer were not significant predictors (all *P* > 0.05) (Supplementary Table 1).

**TABLE 2:** Diagnostic performance of individual biomarkers and combined nomogram for predicting stage iv ovarian endometriotic cysts in training and test cohorts.

Cohort	Parameters	AUC	95% CI	SPE	SEN	NPV	PPV
Training	PLR	0.77	0.69–0.85	0.60	0.83	0.86	0.54
	FIB	0.68	0.59–0.77	0.65	0.67	0.78	0.51
	CA125	0.70	0.61–0.79	0.53	0.81	0.83	0.49
	Nomogram	0.81	0.75–0.88	0.61	0.87	0.89	0.56
Test	PLR	0.73	0.60–0.87	0.92	0.48	0.77	0.77
	FIB	0.63	0.47–0.78	0.95	0.29	0.71	0.75
	CA125	0.67	0.53–0.81	0.62	0.71	0.80	0.50
	Nomogram	0.78	0.65–0.91	0.90	0.62	0.81	0.76

AUC, area under the receiver operating characteristic curve; CI, confidence interval; NPV, negative predictive value; PPV, positive predictive value; SEN, sensitivity; SPE, specificity.

**FIGURE 2:**
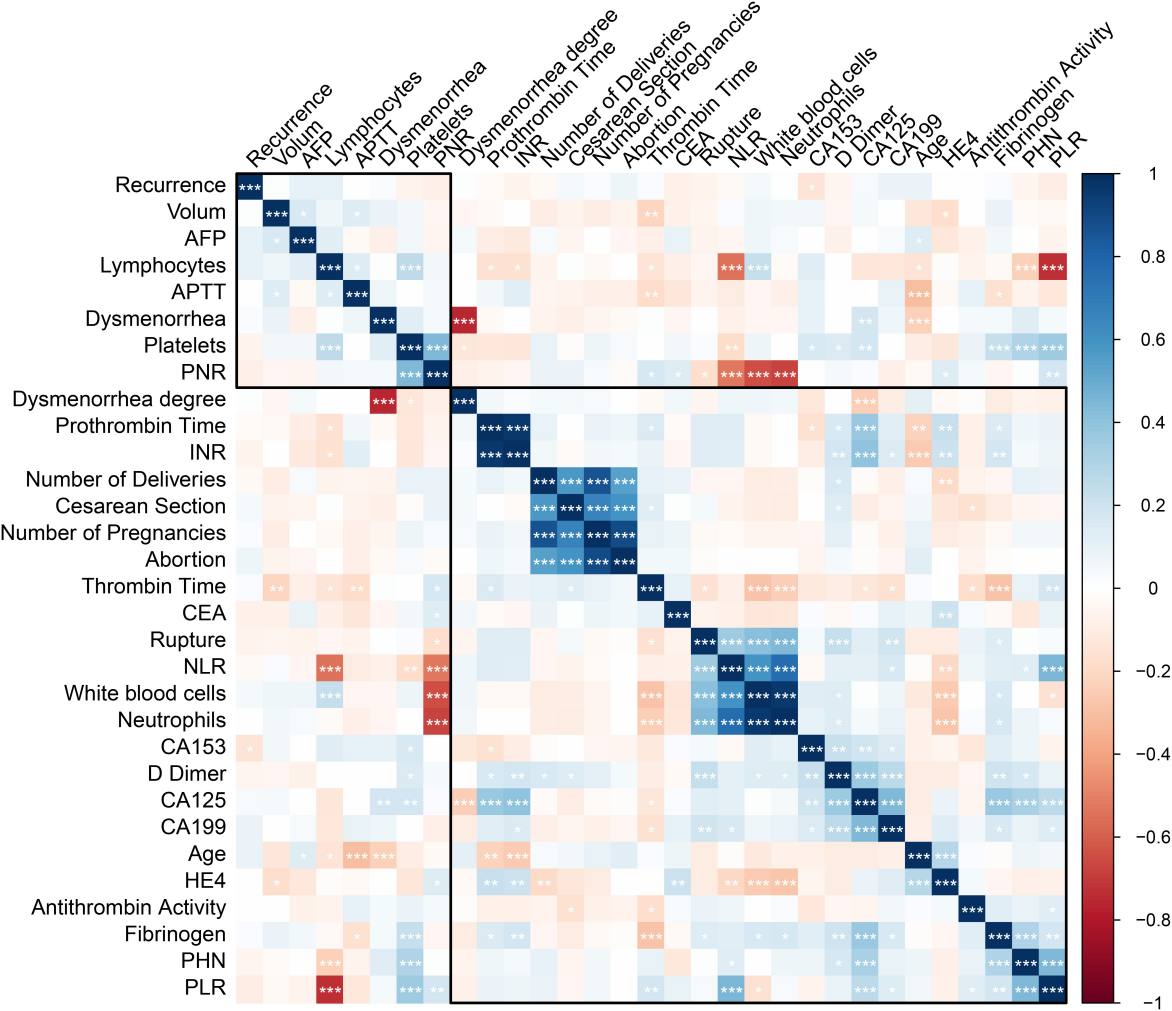
Correlation analysis between disease stage and key biomarkers in ovarian endometriotic cysts (OEC). Spearman correlation analysis examining the relationships between OEC stage and biomarkers in the training cohort. *<0.05; **<0.01; ***<0.001.

Spearman correlation analysis further revealed positive correlations between stage IV OEC and PLR (*r* = 0.44, *P* < 0.001), FIB (*r* = 0.30, *P* < 0.001), and CA125 (*r* = 0.32, *P* < 0.001) (Figure 2).

### Diagnostic efficacy of single and combined biomarkers

**FIGURE 3:**
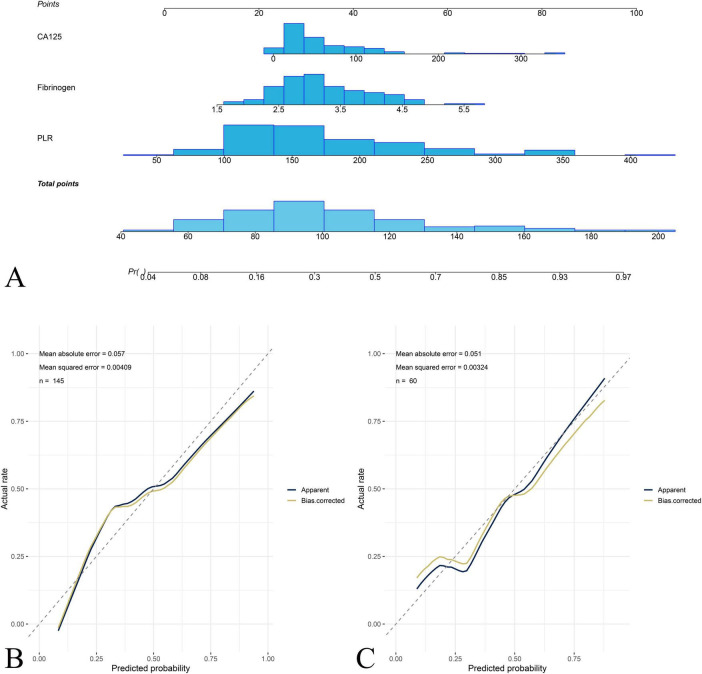
Nomogram construction and calibration for predicting stage IV OEC. **(A)** The nomogram integrating platelet-to-lymphocyte ratio (PLR), fibrinogen (FIB), and carbohydrate antigen 125 (CA125) for predicting the probability of stage IV OEC. Each predictor is assigned a point value based on its contribution to the prediction model, and the total points correspond to the predicted probability of stage IV OEC. The calibration curves demonstrate the agreement between nomogram-predicted probabilities and actual observed frequencies of stage IV disease in the training cohort **(B)** and test cohort **(C)**.

**FIGURE 4:**
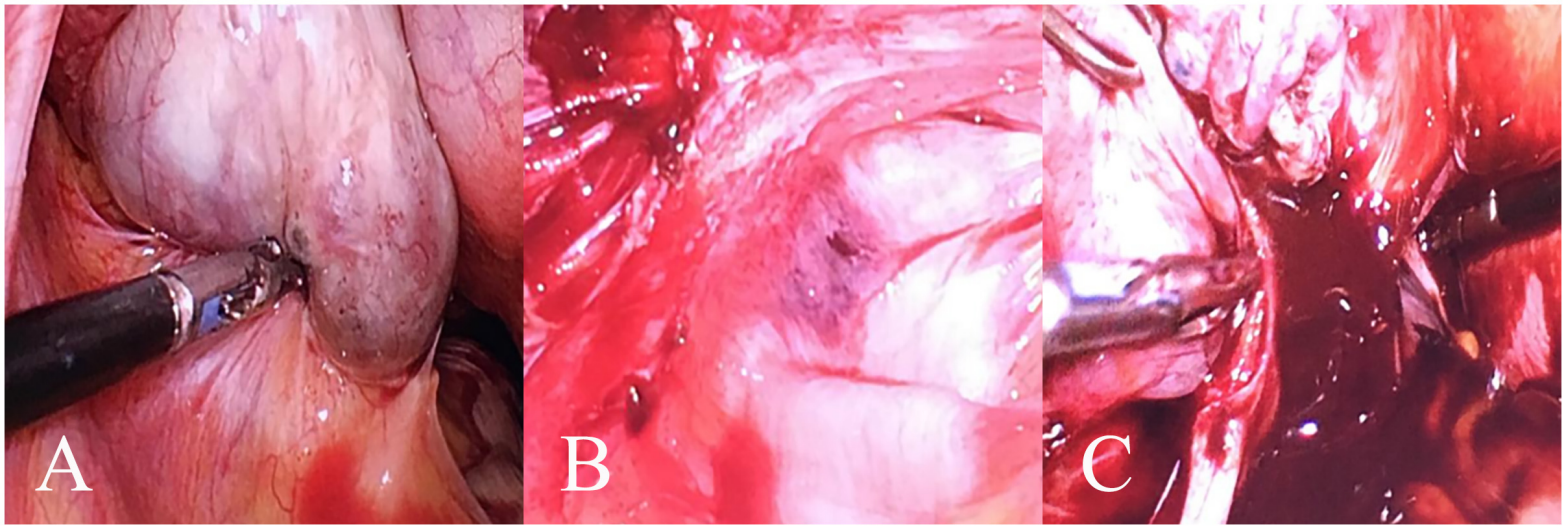
A case of a 49-year-old female patient with left stage IV OEC. In this representative case, the patient’s PLR, FIB, and CA125 values corresponded to a total nomogram score of 215 points, predicting an 83% probability of stage IV disease, which was subsequently confirmed by histopathology. **(A)** Purplish-blue endometriotic nodules visible on the ovarian surface. **(B)** Extensive pelvic adhesions involving the bilateral sacrouterine ligaments, lateral pelvic walls, and posterior cul-de-sac, showing the hemorrhagic and fibrotic nature of advanced endometriosi. **(C)** Chocolate-like viscous fluid characteristic of endometriotic cyst contents observed flowing out following cyst rupture during surgical excision.

ROC analysis was performed to assess the diagnostic efficiency of PLR, FIB, and CA125 for predicting stage IV OEC (Table 2). The nomogram was built by integrating PLR, FIB, and CA125 (Figure 3). A presented case is shown in Figure 4.

In the training cohort, the AUCs for PLR, FIB, and CA125 were 0.77 (95% CI: 0.69–0.85), 0.68 (95% CI: 0.59–0.77), and 0.70 (95% CI: 0.61–0.79), respectively. The corresponding specificities were 0.60, 0.65, and 0.53, while sensitivities were 0.83, 0.67, and 0.81, respectively. The combined nomogram integrating PLR, FIB, and CA125 achieved the highest predictive accuracy, with an AUC of 0.81 (95% CI: 0.75–0.88), specificity of 0.61, sensitivity of 0.87, negative predictive value (NPV) of 0.89, and positive predictive value (PPV) of 0.56.

In the test cohort, PLR, FIB, and CA125 achieved AUCs of 0.73 (95% CI: 0.60–0.87), 0.63 (95% CI: 0.47–0.78), and 0.67 (95% CI: 0.53–0.81), respectively. The nomogram demonstrated robust external performance with an AUC of 0.78 (95% CI: 0.65–0.91), specificity of 0.90, sensitivity of 0.62, NPV of 0.81, and PPV of 0.76.

### Nomogram calibration and clinical usefulness

**FIGURE 5:**
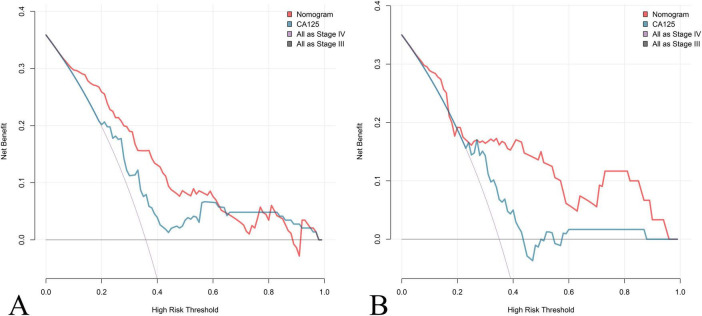
Clinical decision curve (CDC) Analysis of the Nomogram for Predicting Stage IV OEC. CDC evaluates the clinical utility and net benefit of the nomogram for predicting stage IV OEC across different threshold probabilities in both the training cohort **(A)** and test cohort **(B)**. The decision curves compare the net benefit of using the nomogram (red line) against CA125 (blue line) and two reference strategies: treating all patients as stage IV (gray line) and treating no patients as stage IV (horizontal line at zero). The nomogram demonstrates positive net benefit across a wide range of threshold probabilities in both cohorts, indicating that its use for clinical decision-making would result in better outcomes.

Calibration plots for both cohorts demonstrated excellent agreement between predicted and observed probabilities of stage IV OEC (Figure 4). CDC results showed that the nomogram for predicting stage IV OEC added net benefit in both the training and test cohorts (Figure 5).

## Discussion

This study evaluated the diagnostic value of serum inflammatory, coagulation, and tumor markers in differentiating stage III from stage IV OEC. The findings demonstrate that stage IV OEC patients exhibit significantly elevated systemic inflammatory responses, altered coagulation profiles, and increased tumor marker levels. Importantly, the combined analysis of PLR, FIB, and CA125 substantially improved diagnostic sensitivity and accuracy, suggesting potential clinical utility as a non-invasive biomarker panel for stage IV OEC. Figure 1 illustrates the study workflow and patient selection, providing a visual summary of inclusion and exclusion criteria and the randomization into training and test cohorts.

Endometriosis is widely recognized as a chronic inflammatory condition. In our study, stage IV OEC patients showed decreased lymphocyte counts and elevated platelet counts. The findings showed significant difference in PLR between stage III and IV OEC patients. This result is in accordance with the report by Duan et al. (10), who found a positive correlation between PLR and endometriosis in a large cohort of 10,458 Chinese patients of reproductive age. Similarly, Yavuzcan et al. reported significantly elevated PLR values in endometriosis patients compared to healthy controls, suggesting PLR as a potential inflammatory marker (11). Viganò et al. (7) also demonstrated that PLR was significantly higher in women with endometriosis and correlated with disease severity. Previous studies also reported that PLR reflects systemic inflammation and correlates with pelvic adhesion severity in endometriosis (12). Figure 2 provides a graphical representation of the correlation analysis between disease stage and key biomarkers (PLR, FIB, and CA125).

Endometriosis has been associated with abnormal coagulation and fibrinolysis, potentially contributing to lesion development and adhesion formation (13). The results of this study showed that FIB increased significantly with disease progression and correlated positively with rectouterine pouch closure. This suggests that localized coagulation activation may contribute to fibrosis and adhesion formation in advanced disease. Previous studies found that elevated FIB levels in endometriosis patients, which indicates a hypercoagulable state in OEC patients. Fini et al. similarly found elevated FIB and proposed that hyperfibrinogenemia contributes to adhesion formation through increased fibrin deposition (14). The observed positive correlation between FIB levels and rectouterine pouch closure suggests that localized coagulation activity may be linked to adhesion formation and disease progression. These findings indicate that fibrinogen may serve as a marker of disease severity and fibrotic activity rather than a general diagnostic marker for OEC. Further research with broader coagulation parameters is needed to clarify the pathophysiological role of coagulation in OEC. Notably, FIB is a well-established acute phase reactant synthesized primarily by hepatocytes in response to inflammatory cytokines. The relatively modest elevation observed in our stage IV patients could therefore reflect chronic low-grade systemic inflammation associated with advanced endometriosis. Integrating PLR, FIB, and CA125 yields good agreement between predicted and observed probabilities for stage IV OEC in both the training and test cohorts. The calibration curves show near-diagonal alignment, supporting the reliability of the model.

Consistent with prior research, serum CA125 levels were significantly higher in OEC, and both increased with disease severity (15). Elevated CA125 levels correlated with cyst size, rectouterine pouch closure, pelvic adhesions, and dysmenorrhea severity, highlighting its close association with inflammatory and fibrotic processes. Our findings align with previous studies (16), who reported CA125 sensitivity of 52% and specificity of 93% for detecting endometriosis. These results reflect that CA125 levels are more markedly elevated reflecting both disease presence and extent (17, 18). Although CA125 remains the most widely used biomarker for endometriosis, its limited specificity-due to elevation in various conditions including menstruation, pelvic inflammatory disease, and ovarian malignancies-reduces its standalone reliability (19). A previous study recommended combining CA125 with NLR for multi-marker diagnostic approaches (20). This combination represents a practical, cost-effective strategy for improving preoperative diagnostic accuracy in staging OEC. While the mean CA125 levels in ovarian cancer are typically much higher, there is substantial overlap between early-stage cancers and borderline tumors. This underscores why our nomogram is intended for use after endometriosis has been confirmed, not as a diagnostic tool to distinguish OEC from malignancy.

In clinical practice, transvaginal ultrasound and MRI remain the cornerstones of preoperative assessment for ovarian endometriotic cysts; however, both methods primarily reflect structural features and may underestimate the extent of inflammatory fibrosis and adhesion severity that define stage IV disease. Our findings demonstrate that combining CA125 with PLR and FIB markedly enhances sensitivity and diagnostic accuracy, supporting the use of multi-marker models in clinical assessment of OEC staging. The combined assessment (the nomogram) offers a practical and non-invasive approach for distinguishing stage III from stage IV OEC. These markers are inexpensive, routinely available, and easily measurable in clinical laboratories, making them well-suited for preoperative evaluation. Furthermore, their correlation with disease severity and adhesion formation may assist in pre-surgical planning and prognostic assessment. The nomogram addresses a critical gap in current preoperative assessment. The nomogram provides complementary information that enhances imaging-based assessment. The integration of this nomogram into clinical practice could potentially improve rates of successful fertility-sparing surgery. Additionally, earlier and more targeted involvement of reproductive endocrinologists for high-risk patients may reduce the time to conception, which is particularly important given that fertility potential declines with repeated surgeries and disease recurrence. CDC analysis demonstrates that the nomogram confers greater net clinical benefit across a wide range of threshold probabilities than either CA125 alone or default strategies, which substantiates the nomograms real-world utility, showing that its use could improve clinical decision-making by accurately identifying high-risk patients for surgical planning and fertility counseling.

Several limitations should be noted. First, the absence of a healthy control group precluded direct assessment of baseline inflammatory and coagulation states. And all OEC patients in this cohort were in advanced stages (III-IV), limiting the ability to assess biomarker trends in early-stage disease. Second, the class imbalance and small absolute numbers affect interpretation of our results. Given the large number of univariate comparisons, the potential for type I error inflation cannot be completely excluded. Sensitivity analyses such as bootstrapping or repeated random sampling were not performed, which may limit model stability assessment and reproducibility. Future confirmatory studies should consider correction for multiple testing. Third, as a single-center retrospective study, potential selection and measurement biases cannot be fully excluded, which limit the generalizability. Future multicenter prospective studies with both broader disease staging and healthy controls and potential integration with imaging biomarkers are necessary to validate these findings and establish standardized diagnostic thresholds. Moreover, mechanistic studies exploring the link between inflammatory-coagulative pathways and fibrosis in advanced endometriosis may deepen our understanding of disease progression and therapeutic targets.

## Conclusion

The present study demonstrates that integrating PLR, FIB, and CA125 enhances the accuracy of differentiating stage III from stage IV OEC. This combined model provides a practical, non-invasive, and cost-effective tool that can improve preoperative assessment and surgical planning. However, to further strengthen these conclusions, future research should validate these findings in larger, multicenter prospective cohorts and across different populations.

## Data Availability

The raw data supporting the conclusions of this article will be made available by the author, without undue reservation.
