# Supplementary material for: The diagnostic value of serum inflammation, coagulation and tumor indexes in differentiating stage III and IV ovarian endometriotic cysts
Source: Front Med (Lausanne). 2026 Jan 12;12:1733063. doi: 10.3389/fmed.2025.1733063 (PMC12832870; doi:10.3389/fmed.2025.1733063)
Supplement: Supplementary file 1 [file Table_1.docx]

Supplementary Table 1. Results of the multivariate logistic regression

| Parameters | OR | 95%CI | P-value |
| --- | --- | --- | --- |
| (Intercept) | 0.37 | 0.20-0.71 | 0.003 |
| Age | 1.01 | 1.00-1.01 | 0.147 |
| Lymphocytes | 1.03 | 0.78-1.37 | 0.830 |
| Platelets | 1.00 | 1.00-1.00 | 0.432 |
| NLR | 0.99 | 0.96-1.02 | 0.595 |
| PLR | 1.05 | 1.01-1.10 | 0.023 |
| Fibrinogen | 1.11 | 1.02-1.21 | 0.019 |
| D-dimer | 1.13 | 0.84-1.54 | 0.417 |
| CA125 | 1.03 | 1.00-1.06 | 0.036 |

CA125, carbohydrate antigen 125; CI, Confidence Interval; NLR, neutrophil-to-lymphocyte ratio; OR, odds ratio; PLR, platelet-to-lymphocyte ratio
